# CULTURAL ADAPTATION: THE POWER OF COMMUNITY VOICE IN DESIRING A DEMENTIA SUPPORT INTERVENTION FOR LATINE CAREGIVERS

**DOI:** 10.1093/geroni/igae098.3339

**Published:** 2024-12-31

**Authors:** Quinton Cotton, Paul Kissell, Elma Johnson, Manka Nkimbeng, Joseph Gaugler

**Affiliations:** University of Minnesota, Minneapolis, Minnesota, United States; University of Minnesota School of Public Health, Minneapolis, Minnesota, United States; University of Minnesota, Minneapolis, Minnesota, United States; University of Minnesota, Minneapolis, Minnesota, United States; University of Minnesota, Minneapolis, Minnesota, United States

## Abstract

**Intro:**

Latine family caregivers of persons living with Alzheimer’s disease and other related dementias (ADRD) need access to culturally congruent support services. Interventions incorporating community engagement have shown effectiveness in providing ADRD-specific education, supporting caregivers, and mitigating accessibility concerns (transportation and meeting occupational demands).

**Methods:**

Data was collected via structured conversations (World Cafe style discussions) with youth, adult caregivers, and older adults (n=52) on their perspectives, needs, and preferences about aging and dementia needs in the community. Data were coded deductively and analyzed via directed content analysis.

**Results:**

Latine community members shared their interest in developing a community-engaged program that addressed training on health conditions, emphasized the importance of patience and compassion in dementia caregiving, and language concerns. Content analysis revealed community youth emphasizing interpersonal connection and integration of older adults with the broader community; adult caregivers suggesting a need for caregiver self-care and the mitigation of daily scheduling conflicts; and older adults emphasizing need for emotional support, respectful communication, and language barriers in medical, occupational, and service environments. Implications: Community-engaged feedback is vital in developing interventions reflecting Latine community needs and cultural values. Culturally responsive interventions are necessary because they integrate community member experiences and needs while concurrently promoting community ownership over the research process.

